# Do funding sources complement or substitute? Examining the impact of cancer research publications

**DOI:** 10.1002/asi.24726

**Published:** 2022-11-19

**Authors:** Daniele Rotolo, Michael Hopkins, Nicola Grassano

**Affiliations:** ^1^ Science Policy Research Unit (SPRU) University of Sussex Business School Brighton UK; ^2^ Department of Mechanics, Mathematics and Management Polytechnic University of Bari Bari Italy; ^3^ European Commission Joint Research Centre Seville Spain

## Abstract

Academic research often draws on multiple funding sources. This paper investigates whether complementarity or substitutability emerges when different types of funding are used. Scholars have examined this phenomenon at the university and scientist levels, but not at the publication level. This gap is significant since acknowledgement sections in scientific papers indicate publications are often supported by multiple funding sources. To address this gap, we examine the extent to which different funding types are jointly used in publications, and to what extent certain combinations of funding are associated with higher academic impact (citation count). We focus on three types of funding accessed by UK‐based researchers: national, international, and industry. The analysis builds on data extracted from all UK cancer‐related publications in 2011, thus providing a 10‐year citation window. Findings indicate that, although there is complementarity between national and international funding in terms of their co‐occurrence (where these are acknowledged in the same publication), when we evaluate funding complementarity in relation to academic impact (we employ the supermodularity framework), we found no evidence of such a relationship. Rather, our results suggest substitutability between national and international funding. We also observe substitutability between international and industry funding.

## 1 INTRODUCTION

Research funding systems have been the subject of much scrutiny in recent decades. Pressures on national science budgets resulted from the 2007–2008 financial crisis, budget erosion due to inflation and, more recently, the demands of the COVID‐19 pandemic (Boadi, 2014; Murray et al., 2011; Wallace, 2020). These pressures have been reinforced by growing demand to fund research that is more likely to generate impact beyond the academic sphere (de Boer et al., 2007; Lewis, 2015) and is more aligned with societal needs (Ciarli & Ràfols, 2019; Confraria & Wang, 2020). Within this context, policymakers have pursued a major restructuring of national research funding systems. A key principle of these efforts has been the use of “competition for funding” with expectations that competition can improve the system‐level efficiency through the generation of outcome incentives. As a result, major national research evaluation exercises have been established (Edler et al., 2012; Martin, 2011) and external/extramural funding has rapidly increased while internal/intramural funding has reduced (de Boer et al., 2007; Herbst, 2007; Hicks, 2012; Lewis, 2015). Although a positive impact of funding is observed on the productivity of research organizations (Bolli & Somogyi, 2011; Cattaneo et al., 2016) and scientists (Jacob & Lefgren, 2011; Lee & Bozeman, 2005), the evidence of research system‐level productivity gains as a result of higher levels of competition in the allocation of research funding are mixed (Auranen & Nieminen, 2010; Himanen et al., 2009). Within this context, our understanding of the interdependencies that characterize funding environments remains limited. Funding systems are populated by a large variety of organizations often playing different roles, funding distinct types of research, but also these organizations are strongly interdependent on other funding efforts to maximize the benefit of the research they support. This complexity poses major challenges to the governance of funding systems. Understanding if synergies emerge between funding of different types can provide insights to develop more efficient and effective allocation mechanisms of the resources for research.

A limited number of studies have delved into the phenomenon of complementarity or substitutability between funding sources, focusing on research organizations and scientists as units of analysis. Such research has provided seminal evidence that public and private funding sources complement each other at the university level since these sources are likely to co‐occur in supporting university research (e.g., Blume‐Kohout et al., 2009; Muscio et al., 2013; Payne, 2001). In contrast, at the individual scientist level, research has suggested that complementarity exists between government and industry funding sources, while other combinations of sources (e.g., government and foundation grants) are substitutes (Grimpe, 2012; Hottenrott & Richstein, 2020). However, the role of combinations of funding sources in supporting the intense collective research effort required to produce a single piece of research, that is, a scientific publication, has been overlooked. Scientific publications are often supported by more than one funding source (e.g., Álvarez‐Bornstein et al., 2017; Rigby, 2013; Yan et al., 2018). A grant‐funded research project can result in a publication output that has been supported by multiple funding sources. These sources may be accessed directly by the original team of researchers in the project and/or indirectly through collaboration with researchers external to this original project team, who bring their own resources to joint publications. On this basis, we argue that funding complementarity and substitutability can emerge at the level of a single research outputs. Yet, our understanding of this phenomenon is limited.

We aim to address this gap by examining whether external funding (i.e., funding for research obtained from beyond the researchers' own institutions) of different types are complements or substitutes when considering scientific publications as the unit of analysis. We first assess funding complementarity and substitutability by examining the likelihood of different funding types to support jointly a publication (co‐occurrence). We then build on the supermodularity theory (Milgrom & Roberts, 1995) to examine funding complementarity and substitutability in relation to publications' academic impact (citation count). Accordingly, two funding sources are complements (substitutes) if the marginal academic impact that is associated with their joint use in a publication is larger (smaller) than the marginal academic impact that is associated with their use in isolation. This approach enables us to examine complementarity and substitutability beyond the accumulation of funding, hence, to assess the emergence (if any) of synergistic interactions between different types of funding.

Our empirical analysis relies on a sample of cancer‐related publications from the year 2011 by scientists based in research organizations in the United Kingdom. This sample provides the opportunity to assess the academic impact of publications after citations have accumulated for around 10 years. We identify the funding sources of these publications by examining funding disclosures reported in acknowledgment sections (e.g., Cronin, 1991; Lewison & Dawson, 1998; Rigby, 2013). We distinguish three main types of external funding sources: (a) national funding (i.e., support from funders based in the United Kingdom), (b) international funding (i.e., support from funders based outside the United Kingdom), and (c) industry funding. The econometric analysis suggests the emergence of complementarity between funding types—in particular between national and international funding—only in terms of co‐occurrence in funding a publication. Our findings, in contrast, reveal substitutability between national and international funding, and international and industry support when the relationship between these combinations of funding types and the academic impact of the publications they support is examined.

The remainder of the paper is organized as follows. Section 2 reviews major studies examining the role of funding in research systems and the issue of funding complementarity and substitutability. In this section, we also present the conceptualization of complementarity and substitutability that underlies our empirical approach. Section 3 introduces the research setting, the data, and the methodology of our study. Section 4 presents the results, while Section 5 concludes the paper by discussing the implications of our study, its limitations, and future research opportunities.

## 2 THEORETICAL BACKGROUND

### 2.1 Research funding: Systems, impact, and collaboration

Over the last few decades, the governance of science, the delineation of research priorities, and funding mechanisms have significantly changed from a centralized approach to more competitive models. These changes have aimed to respond to budget constraints, demands for greater accountability, and the need to generate research outcomes of societal value (e.g., de Boer et al., 2007; Leisytë & Kizniene, 2006; Lewis, 2015; Whitley & Gläser, 2007). As a result, the percentage of core/block funding—i.e., funding that the State allocates to research organizations—has reduced in favor of competitive project‐based funding (e.g., Auranen & Nieminen, 2010; Herbst, 2007; Hicks, 2012). This allocation model is expected to improve the efficiencies of funding systems since resources are given to the “top performers” and stronger incentives for scientists and research organizations exist to produce research outcomes that achieve societal goals. Research is therefore undertaken in “quasi‐market” conditions since research actors compete and access (also through collaboration) different funding sources. This trend has resulted in an increasing emphasis on the evaluation of research outputs, thus driving the emergence of metrics and major national evaluation programs (Edler et al., 2012; Martin, 2011; Reed et al., 2021; Wilsdon et al., 2015).

The rising use of competitive funding models has triggered a substantial academic and policy debate (e.g., Geuna, 2001; Geuna & Martin, 2003; Hicks, 2012; Orr et al., 2007). However, there is no conclusive evidence that more competition in allocating financial resources leads research systems to superior efficiency in terms of research output (e.g., Auranen & Nieminen, 2010; Butler, 2003; Himanen et al., 2009). The priorities of funders as well as the different emphasis that national exercises of research evaluation have placed on bibliometric‐based indicators of research quantity and quality, or on the impact of research beyond the academic context, have been very influential on research outcomes and practice (Civera et al., 2020; Rebora & Turri, 2013; Zacharewicz et al., 2019). In this regard, questions have been raised on whether too much competition could be disruptive to research activity (e.g., conducting research vs. writing project proposals, publication inflation) as well as to the ability of the review process to assess the quality of publications and grant applications (Bornmann et al., 2010; Kaplan et al., 2008). Within this stream of research, evidence has converged on a positive relationship between various types of funding sources and the productivity of universities (Bolli & Somogyi, 2011; Bonaccorsi et al., 2006; Cattaneo et al., 2016; Payne & Siow, 2003) and individual scientists (Defazio et al., 2009; Gulbrandsen & Smeby, 2005; Jacob & Lefgren, 2011; Kelchtermans & Veugelers, 2011; Lee & Bozeman, 2005; Van Looy et al., 2004). Similarly, funding has been found to be positively related to collaboration (Adams et al., 2005; Bozeman & Corley, 2004; Ubfal & Maffioli, 2011).

### 2.2 Research funding: Complementarity and substitutability

Drawing on the literature examining the relationship between public and private funding to research (e.g., David et al., 2000; David & Hall, 2000), scholars have examined complementarity and substitutability between different research funding sources by considering two units of analysis: universities and scientists.

University‐level research has outlined the presence of complementarity effects between different types of funding sources in terms of co‐occurrence of sources: there is a positive relationship between the funding a university allocates or receives for research and other types of financial resources. For example, Connolly (1997) examined the internal and external funding of 195 U.S. universities from 1979 to 1990 and found a positive correlation between internal support for research (i.e., funding that is budgeted for research by universities and provided to their researchers) and funding that universities received from external sponsors (e.g., government, industry, nonprofit organizations). In the same vein, Payne (2001) found complementarity between federal funding to U.S. research universities and private donations, where “[…] on average, increasing federal research funding by $1 increases private donations to research universities by approximately 65 cents […]” (p. 749). Blume‐Kohout et al. (2009) provided similar evidence of complementarity by examining federal and nonfederal funding in life sciences for U.S. universities, while Muscio et al. (2013) found that government funding to Italian universities complements funding received from private actors in terms of research contracts and consulting activities. This suggests that external sponsors can interpret internal/public funding as a signal of research quality, an argument that is also supported by recent evidence from Bonaccorsi et al. (2021) on the positive relationship between research quality and its valorization (i.e., patents and spin‐off companies) in the case of the Italian Evaluation of Research Quality VQR (2011–2014).

While at the organizational level there is no evidence of substitutability between funding sources, studies examining this phenomenon at the level of individual scientists have provided mixed results. For example, Grimpe (2012) examined the funding sources received by scientists at German universities and public research institutes and found complementarity between government and industry funding since these sources are likely to be jointly awarded to scientists. However, the study also provided evidence of substitutability between government and foundation grants, between funding from EU Framework Program 6 and foundations, and between EU Framework Program 6 and industry support. In the same vein, Hottenrott and Lawson (2017) examined complementarity and substitutability between public and industry funding in relation to research quality (publication count, citations, and average impact factors) and outcomes (patentability) in the case of academic scientists based at UK universities. This analysis provided evidence of complementarity between public and private funding only with the increase of a scientist's patentability of her/his research outcome.

Examining complementarity and substitutability at the levels of universities and scientists provides inevitably a partial perspective on the interaction among different funding sources. Such an approach neglects the continuously expanding research collaboration networks (e.g., De Solla Price, 1963; Fanelli & Larivière, 2016; Wuchty et al., 2007). Research often involves more than one academic with their own associated funding sources, spread across multiple research organizations. The complementarity or substitutability of these funding sources can occur at the level of the single research output. Yet knowledge of these interactions remains scant. Understanding how different types of funding sources co‐occur to support research and any potential synergy that may arise from this remains critical to develop policies capable of distributing resources more effectively and efficiently as well as to assess the impact of potential cuts to research budgets.

Our paper contributes to this literature by investigating complementarity and substitutability between different types of funding in the production of a single piece of research, that is, a publication. We examine this phenomenon in two ways. First, we analyze the extent to which different types of funding sources are likely to co‐occur in supporting a publication conditional to other publication‐level characteristics (e.g., number of authors/affiliations, research domain, type of research output). The fact that two funding types are jointly used to produce a publication indicates that the publication might not exist without the co‐occurrence of these sources. Second, we examine complementarity and substitutability in relation to performance, in particular the academic impact of a publication. To do so, we build on Milgrom and Roberts's (1995) supermodularity theory and its formulation of synergies and system effects according to which the “whole is more than the sum of its parts” (see also Ennen & Richter, 2010). More precisely, we consider the use of two types of funding sources, A and B, and their relationship with the performance Π, namely the academic impact of a publication, as $\Pi \left(A,B\right)$—this will be operationalized as the logarithm of the number of citations the publication received as we will elaborate later in the paper. If the publication is supported by the funding type A then A=1, otherwise A=0; similarly for B. A and B are complements (supermodular) if $\Pi \left(1,1\right)-\Pi \left(0,1\right)\geq \Pi \left(1,0\right)-\Pi \left(0,0\right)$; they are substitutes (submodular) if $\Pi \left(1,1\right)-\Pi \left(0,1\right)\leq \Pi \left(1,0\right)-\Pi \left(0,0\right)$. In other words, ceteris paribus, there is complementarity (substitutability) between two funding types A and B if the marginal difference of a publication's academic impact that is associated with adding the source A—i.e., $\left[\Pi \left(1,1\right)-\Pi \left(0,1\right)\right]$—while the source B is also used is larger (smaller) than the marginal difference of the publication's academic impact associated with the use of the funding type A in isolation—i.e., $\left[\Pi \left(1,0\right)-\Pi \left(0,0\right)\right]$—and vice versa.^1^

Following the lead of previous research (e.g., Athey & Stern, 1998; Fares et al., 2018), we refer to the latter empirical strategy to examine complementarity and substitutability as the “direct approach” since the assessment of complementarity and substitutability is against a given measure of performance, while we refer to the former empirical strategy (co‐occurrence) as the “indirect approach.” We will elaborate on the operationalization of these approaches in the next section.

## 3 DATA AND METHODOLOGY

### 3.1 Research setting and data

We examine funding complementarity and substitutability in cancer research produced by scientists employed in UK research organizations in 2011. The selection of data from the year 2011 allows us to evaluate publications' academic impact over a period of about 10 years during which citations have accumulated. The focus on cancer research is particularly suitable for the purpose of our study as this area has been characterized by intense funding activity from a variety of sources (e.g., Begum et al., 2018; Lewison & Sullivan, 2015; Schmutz et al., 2019). The UK funding system is populated by a large variety of organizations (from industry to, government, charities, and philanthropic funders), with prominent co‐occurrence of funders supporting research (Grassano et al., 2017).

We map the UK funding landscape for cancer research by using the funding data reported in the acknowledgement sections of cancer‐related publications in the year 2011—previous research has demonstrated that the rate of reporting research funding in acknowledgment sections is relatively high in medical research (e.g., Costas & Van Leeuwen, 2012; Dawson et al., 1998; Lewison, 1998). We consider UK publications as those publications involving at least one research organization based in the UK. We delineated cancer research building on the Medical Subject Heading (MeSH) classification of the MEDLINE/PubMed. More precisely, we captured cancer‐related publications as those assigned to the “Neoplasms” descriptor. This search also included all records assigned to relative sublevels in the tree structure. The initial sample included 115,101 documents published globally in 2011. We matched this dataset with SCOPUS data to retrieve full data on authors' affiliations or research host organizations—we used the MEDLINE/PubMed publication unique identifier (PMID), while we relied on publications' DOI when this was not available (Rotolo & Leydesdorff, 2015). The match covered 98.1% of the initial sample of publications. We then identified UK publications as those involving at least one author affiliated with a UK research organization. The remaining unmatched records (1.9%) were manually screened and added to the dataset when a UK research organization was found.

The resulting dataset included 7,922 publications, for which we obtained full‐text access for 7,510 records (94.8%). Acknowledgments sections (or similar) in publications provide access to information about any external funding (in addition to the funding authors receive from their affiliations) that supported the research (e.g., Cronin et al., 1992; MacKintosh, 1972; McCain, 1991). In this regard, funding acknowledgments have been used to study the relationship between funding and citation impact (e.g., Gök et al., 2016; Yan et al., 2018). Although funding acknowledgments have been the subject of criticisms in terms of coverage and accuracy (Liu, 2021; Tang et al., 2017), we argue that these limitations are less prominent in our case, given we deal with cancer research published in English by UK‐based authors. For each publication, we searched for all the funding sources that supported the authors and their research organizations to produce the publication—more details on the methodology are available elsewhere (Grassano et al., 2017).

Around 48% of the 7,510 publications in the sample did not disclose a source of funding, while the remaining records reported at least one funder. From this sample, we excluded publications in the top 1% in terms of number of authors—i.e., all publications with more than 45 authors—since these represent outlier cases of research collaboration with potentially a large number of funding sources for which complementarity and substitutability would be challenging to interpret. The final sample includes 7,439 records.

### 3.2 Variables

We first classified external funding sources into three mutually exclusive categories: (a) national (i.e., UK‐based funders excluding industry), (b) international (i.e., non‐UK‐based funders, excluding industry), and (c) industry. We then created our dependent variables. To evaluate what correlates with the likelihood of a publication to be supported by one or more funding sources, we defined a variable counting the number of external funding sources acknowledged in the publication—*External funding source*—and the corresponding dummy variable—*External funding source (d)*. Subsequently, we created three dummy variables for each category of external funding—*National funding (d)*, *International funding (d)*, *Industry funding (d)*—and the variable *Citations*, that is, the natural logarithm of the number of citations publications received plus one, as a proxy for academic impact. We considered the logarithm of the number of citations a publication receives to compensate for censoring issues, that is, for the fact that a publication will continue to be cited after its citations are counted (Stringer et al., 2010).^2^

We included a number of control variables in our models. First, we controlled for the variety of research domains of a publication—*Research variety*—counting the number of distinct MeSH qualifiers assigned to the publication. Qualifiers are part of the MeSH classification system and further specify the content of publications in relation to the descriptor to which they are assigned. Second, we controlled for the extent to which the research is focalized on specific aspects of cancer—*Research specificity*—as the lowest level of the “Neoplasms” branch of the MeSH tree assigned to a publication. Third, we included two dummy variables—*Article (d)* and *Review (d)*—assuming value one if a publication is an article or a review, respectively. Fourth, we controlled for the *Number of references* and *Number of authors*. Fifth, some publications involve more international organizations than others. To avoid collinearity issues with the variable *Number of authors*, we controlled for the number of distinct countries involved in a publication as listed in authors' affiliation addresses out of the number of authors—*Internationality ratio*. Sixth, and similarly, we controlled for the number of distinct affiliations involved in a publication as listed in authors' affiliation addresses out of the number of authors—*Affiliation ratio*. Seventh, we controlled for major research domains by including 18 dummy variables for those MeSH qualifiers assigned to at least 5% of the publications in our sample. Finally, we included 26 dummy variables for publications involving the most productive research organizations in our sample as those contributing to at least 1% of the total research output, and 14 dummy variables for the top 1% journals by number of publications.^3^ All variables are described in Table 1.

**TABLE 1:** Descriptions of the variables

Variable	Description
External funding	Number of external funding sources acknowledged in a publication
External funding (d)	Dummy variable set to one if at least one external funding source is acknowledged in a publication
National funding (d)	Dummy variable set to one if at least one UK‐based funding source is acknowledged in a publication
International funding (d)	Dummy variable set to one if at least one non‐UK‐based funding source is acknowledged in a publication
Industry funding (d)	Dummy variable set to one if at least one industry funding source is acknowledged in a publication
Citations	Natural logarithm of the number of citations a publication received plus one from the publication date to July 24, 2021
Research variety	Number of distinct MeSH qualifiers assigned to a publication
Research specificity	Lowest level of the MeSH C04 (neoplasms) descriptors assigned to a publication
Article (d)	Dummy variable set to one if the publication is an article
Review (d)	Dummy variable set to one if the publication is a review
Number of references	Number of references cited in the publication
Number of authors	Number of authors listed in the publication
Internationality ratio	Number of distinct countries involved in a publication as reported in affiliation addresses divided by the number of authors listed in the publication
Affiliations ratio	Number of distinct affiliations involved in a publication as reported in affiliation addresses divided by the number of authors listed in the publication
Research domains (d)	Dummy variables for research domains: blood, complications, drug therapy, epidemiology, etiology, genetics, immunology, metabolism, mortality, pathology, physiopathology, prevention and control, radiography, radiotherapy, secondary, surgery, and therapy (18 dummy variables)
Research organizations (d)	Dummy variables for the UK research organizations that contributed to at least 1% of the publication sample (26 dummy variables)
Journals (d)	Dummy variables for the top 1% journals by number of publications in the sample (14 dummy variables)

*Note*: (d) refers to dummy variables.

*Source*: Authors' elaboration.

### 3.3 Empirical strategy and model specification

We first adopted the “indirect approach” to investigate funding complementarity and substitutability (Athey & Stern, 1998). To do so, we examined to what extent funding types co‐occur in publications conditional to other publication‐level characteristics. Following previous research (Arora & Gambardella, 1990; Fares et al., 2018; Grimpe, 2012; Reichstein & Salter, 2006), we estimated a multivariate probit model (Cappellari & Jenkins, 2003). We considered the following equations:

$$\left\{\begin{array}{l} y_{\mathrm{Nat},i}^{*}=\beta_{\mathrm{Nat}}X_{\mathrm{Nat},i}+\varepsilon_{\mathrm{Nat},i}\\ y_{\mathrm{Int},i}^{*}=\beta_{\mathrm{Int}}X_{\mathrm{Int},i}+\varepsilon_{\mathrm{Int},i}\\ y_{\mathrm{Ind},i}^{*}=\beta_{\mathrm{Ind}}X_{\mathrm{Ind},i}+\varepsilon_{\mathrm{Ind},i} \end{array}\right.,$$

where $y_{\mathrm{Nat},i}^{*}$, $y_{\mathrm{Int},i}^{*}$, and $y_{\mathrm{Ind},i}^{*}$ represent our latent dependent variables assuming value one if the publication i received a certain type of funding source—national, international, and industry funding, respectively—zero otherwise; $\beta_{\left\{\mathrm{Nat}/\mathrm{Int}/\mathrm{Ind}\right\}}$ are the coefficients, $X_{\left\{\mathrm{Nat}/\mathrm{Int}/\mathrm{Ind}\right\},i}$ the covariates, and $\varepsilon_{\left\{\mathrm{Nat}/\mathrm{Int}/\mathrm{Ind}\right\},i}$ the error terms, which are assumed to be distributed as multivariate normal with mean equal to zero and variance–covariance matrix *V*. The variance–covariance matrix *V* is a symmetric matrix with diagonal values equal to one. The multivariate probit estimation returns correlations among the errors terms that provide evidence of the extent to which if a certain type of funding is acknowledged in a publication also another type of funding is acknowledged in the publication, that is, to what extent funding types are complements or substitutes on the basis of their co‐occurrence.

We then examined funding complementarity and substitutability by using a “direct approach,” which considers the number of citations a publication received as a dimension of performance Π. We followed previous research efforts (e.g., Antonioli et al., 2017; Ballot et al., 2015; Cassiman & Veugelers, 2006; Mohnen & Röller, 2005) and estimated the following equation:

$$\begin{array}{c} {\text{Citations}}_{i}=\left\{F_{\mathrm{Nat}}^{i},F_{\mathrm{Int}}^{i},F_{\mathrm{Ind}}^{i},X,\theta ,\beta \right\}\\ =\left(1-F_{\mathrm{Nat}}^{i}\right)\left(1-F_{\mathrm{Int}}^{i}\right)\left(1-F_{\mathrm{Ind}}^{i}\right)\theta_{000}\\ +F_{\mathrm{Nat}}^{i}\left(1-F_{\mathrm{Int}}^{i}\right)\left(1-F_{\mathrm{Ind}}^{i}\right)\theta_{100}+\left(1-F_{\mathrm{Nat}}^{i}\right)F_{\mathrm{Int}}^{i}\left(1-F_{\mathrm{Ind}}^{i}\right)\theta_{010}\\ +\left(1-F_{\mathrm{Nat}}^{i}\right)\left(1-F_{\mathrm{Int}}^{i}\right)F_{\mathrm{Ind}}^{i}\theta_{001}+F_{\mathrm{Nat}}^{I}F_{\mathrm{Int}}^{i}\left(1-F_{\mathrm{Ind}}^{I}\right)\theta_{110}\\ +F_{\mathrm{Nat}}^{i}\left(1-F_{\mathrm{Int}}^{i}\right)F_{\mathrm{Ind}}^{i}\theta_{101}+\left(1-F_{\mathrm{Nat}}^{i}\right)F_{\mathrm{Int}}^{i}F_{\mathrm{Ind}}^{i}\theta_{011}\\ +F_{\mathrm{Nat}}^{i}F_{\mathrm{Int}}^{i}F_{\mathrm{Ind}}^{i}\theta_{111}+X^{i}\beta +\varepsilon^{i}, \end{array}$$

where $F_{\left\{\mathrm{Nat}/\mathrm{Int}/\mathrm{Ind}\right\}}^{i}$ refers to the funding received by publication i (national, international, or industry), thus assuming value one when a type of funding supported the publication, zero otherwise. θ represents the coefficients associated with specific combinations of funding types. X are the remaining covariates, while β the corresponding coefficients.

**FIGURE 1:**
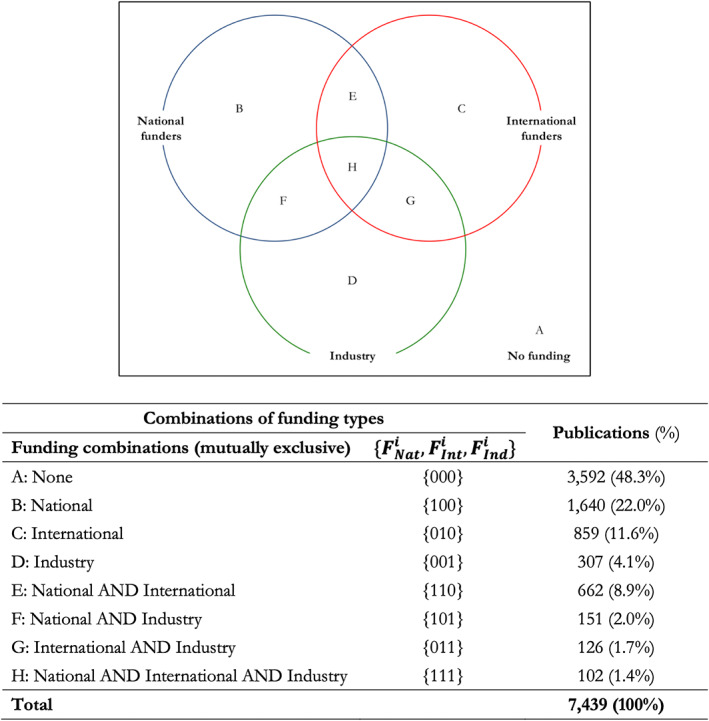
Combinations of funding types in UK cancer publications (*Source*: Authors' elaboration)

Since our analysis includes three types of funding sources, we operationalize the equation with eight mutually exclusive combinations of these sources: F000 assumes value one if a publication does not acknowledge any source of external funding; F100 assumes value one if a publication acknowledges only sources of national funding; F010 assumes value one if a publication acknowledges only sources of international funding; F001 assumes value one if a publication acknowledges only sources of industry funding; F110 assumes value one if a publication acknowledges only sources of national and international funding; F101 assumes value one if a publication acknowledges only sources of national and industry funding; F011 assumes value one if a publication acknowledges only sources of international and industry funding; F111 assumes value one if a publication acknowledges sources of national, international, and industry funding. These combinations are depicted and empirically described in Figure 1. We tested for conditional complementarity and substitutability between each pair of funding types considering the presence and absence of the third type of funding. All econometric models were estimated in Stata 17 with robust standard errors clustered at the journal level. Given the focus on academic impact as the dependent variable, the control variables included in the model and the analytical approach attempt to minimize any potential Matthew Effect on the observed results.^4^

## 4 RESULTS

We first examined the descriptive statistics and correlation matrix of our variables—we found no evidence of major collinearity issues that may affect our econometric estimation—and explored the extent to which publication‐level characteristics (control variables) are associated with the presence and number of funding sources in publications' acknowledgements (see Data S1, Supporting Information).

We then examined funding complementarity and substitutability. We first adopted the “indirect approach” by examining the extent to which pairs of funding types co‐occur in publications conditional on the publication‐level control variables we outlined above. The estimation of the multivariate probit model is reported in Table 2. Publications that were not supported by any external funding represent the benchmark case. The tests for correlation of the errors terms between the three equations associated with the three types of funding provide evidence of a positive correlation between national and international funding (*ρ*21 = 0.099, *p* < .01), thus suggesting complementarity between these two sources of research support conditional on the publication‐level covariates included in the model.

**TABLE 2:** Multivariate probit model: national, international, and industry funding (*N* = 7,439)

Independent variables	(1) Funding		
	National	International	Industry
Research variety	0.006 (0.054)	−0.124* (0.064)	−0.128 (0.089)
Research specificity	−0.050*** (0.010)	−0.040*** (0.011)	−0.000 (0.014)
Article (d)	0.583*** (0.074)	0.638*** (0.086)	0.401*** (0.121)
Review (d)	0.427*** (0.099)	0.144 (0.106)	0.232* (0.130)
Number of references	0.004*** (0.001)	0.005*** (0.001)	0.003*** (0.001)
Number of authors	−0.022*** (0.004)	0.095*** (0.007)	0.055*** (0.004)
Internationality ratio	−0.331** (0.167)	0.772*** (0.136)	0.376** (0.148)
Affiliations ratio	−1.218*** (0.121)	0.207** (0.102)	0.226* (0.119)
Blood (d)	−0.077 (0.137)	−0.185 (0.212)	−0.060 (0.207)
Complications (d)	−0.563*** (0.114)	−0.443*** (0.140)	−0.051 (0.169)
Diagnosis (d)	−0.249*** (0.072)	−0.162* (0.096)	0.037 (0.116)
Drug therapy (d)	−0.176** (0.074)	−0.147 (0.097)	0.854*** (0.105)
Epidemiology (d)	0.166 (0.108)	0.167 (0.110)	−0.035 (0.142)
Etiology (d)	0.046 (0.129)	0.436*** (0.121)	−0.011 (0.176)
Genetics (d)	0.299*** (0.085)	0.443*** (0.091)	−0.068 (0.123)
Immunology (d)	0.139 (0.130)	0.417*** (0.134)	−0.241 (0.220)
Metabolism (d)	0.299*** (0.085)	0.443*** (0.091)	−0.068 (0.123)
Mortality (d)	0.114 (0.119)	0.130 (0.142)	0.011 (0.153)
Pathology (d)	−0.097 (0.070)	0.232*** (0.078)	−0.002 (0.112)
Physiopathology (d)	0.020 (0.158)	0.251 (0.166)	−0.040 (0.228)
Prevention and control (d)	−0.031 (0.132)	0.086 (0.149)	0.359** (0.170)
Radiography (d)	−0.325** (0.128)	−0.270 (0.238)	0.225 (0.243)
Radiotherapy (d)	−0.025 (0.142)	−0.179 (0.231)	0.039 (0.152)
Secondary (d)	−0.386*** (0.127)	−0.597*** (0.181)	0.087 (0.163)
Surgery (d)	−0.603*** (0.086)	−0.586*** (0.122)	−0.237* (0.124)
Therapy (d)	−0.207** (0.090)	−0.226** (0.103)	0.307*** (0.117)
Research organizations (d)	Included	Included	Included
Journals (d)	Included	Included	Included
Constant	−0.438*** (0.115)	−2.189*** (0.124)	−2.557*** (0.162)
*ρ*21, *ρ*31		0.099***	0.022
*ρ*32			0.027
Log‐likelihood	−8602.9		
(Wald)*χ* ^2^	3766.8***		

*Note*: Robust standard errors are clustered at the journal level and are reported in parentheses.

*p < .1; **p < .05; ***p < .01.

*Source*: Authors' elaboration.

The model also outlines a number of relationships between our control variables and the likelihood of a type of funding to be acknowledged in a publication. First, the more research domains a publication focus on (*Research variety*) the less likely the publication is to acknowledge the support of international funding. Second, the more specific are the aspects of cancer that are examined in a publication, the lower is the likelihood that the publication is supported by national and/or international funding. Third, *Articles* and *Reviews* are more likely to report the support of national funding than any other document type. Articles are also more likely to report support from international and industry funding. Forth, the *Number of references* of a publication is positively related to the acknowledgement of any funding source. Fifth, the larger is the research team (*Number of Authors*) involved in a publication the higher is the likelihood that the publication is produced by accessing international and/or industrial support than national funding. Sixth, as one would expect, research teams that are located in a larger number of countries are associated with publications reporting support of international and industry funding rather than national funding. In the same vein, research teams that involve a larger number of research organizations (affiliations) are associated with publications reporting international and industry support rather than national funding.

Finally, when examining publications' research domains, we found that publications in genetics and metabolism are more likely to acknowledge at least one national and/or international funding source. Yet, these domains are not related to industrial support. Similarly, publications on complications, diagnosis, secondary, surgery, and therapy are less likely to be supported by national and/or international funding, while the surgery domain is negatively related to support from industrial funding. The domains of drug therapy, prevention and control, and therapy are the only domains positively associated with the presence of industrial funding. Cancer publications are also less likely to be supported by national funding when in radiography. International funding is positively related to publications in etiology, immunology, and pathology.

We concluded the analysis by examining funding complementarity and substitutability according to the “direct approach.” We built on supermodularity theory and examined funding complementarity and substitutability in relation to the number of citations a publication received. In Table 3, Model 2 provides evidence that national, international, and industry funding are all positively related to the number of citations. In line with previous research (e.g., Gök et al., 2016; Yan et al., 2018), publications that acknowledge research funding are more highly cited by subsequent research. Model 3 tests for the interaction between different funding types. These interaction terms are all highly significant with the exception of the interaction between national and industry funding and are negatively related to a publication's number of citations, thus providing preliminary evidence of the presence of substitutability between the different pairs of funding types as further discussed below. Finally, Model 4 presents the results of the regression analysis when the eight mutually exclusive combinations of funding types are considered. This specification confirms that funding types (in any combination) are positively related to the number of citations publications receive when compared with publications with no funding acknowledgments.^5^

**TABLE 3:** OLS estimation: number of citations and funding types (*N* = 7,439)

Independent variable	(2) Citations (ln)	(3) Citations (ln)	(4) Citations (ln)
National funding (d)	0.324*** (0.034)	0.455*** (0.041)	
International funding (d)	0.234*** (0.036)	0.466*** (0.048)	
Industry funding (d)	0.249*** (0.063)	0.506*** (0.089)	
National funding (d) * International funding (d)		−0.395*** (0.068)	
National funding (d) * Industry funding (d)		0.194* (0.111)	
International funding (d) * Industry funding (d)		−0.540*** (0.105)	
F000 (None)			1.110*** (0.085)
F100 (National)			1.565*** (0.086)
F010 (International)			1.576*** (0.103)
F001 (Industry)			1.616*** (0.123)
F110 (National AND International)			1.636*** (0.100)
F101 (National AND Industry)			1.876*** (0.128)
F011 (International AND Industry)			1.540*** (0.140)
F111 (National AND International AND Industry)			1.409*** (0.155)
Research variety	0.032 (0.043)	0.027 (0.043)	0.027 (0.043)
Research specificity	−0.032*** (0.008)	−0.031*** (0.008)	−0.031*** (0.008)
Article (d)	1.050*** (0.054)	1.014*** (0.053)	1.014*** (0.053)
Review (d)	1.300*** (0.069)	1.287*** (0.069)	1.287*** (0.069)
Number of references	0.010*** (0.001)	0.009*** (0.001)	0.009*** (0.001)
Number of authors	0.058*** (0.007)	0.058*** (0.006)	0.058*** (0.006)
Internationality ratio	−0.184 (0.113)	−0.140 (0.110)	−0.140 (0.110)
Affiliations ratio	0.251*** (0.081)	0.222*** (0.080)	0.222*** (0.080)
Blood (d)	−0.104 (0.111)	−0.094 (0.111)	−0.094 (0.111)
Complications (d)	−0.179** (0.082)	−0.155* (0.081)	−0.155* (0.081)
Diagnosis (d)	−0.164*** (0.057)	−0.155*** (0.057)	−0.155*** (0.057)
Drug therapy (d)	0.078 (0.066)	0.066 (0.065)	0.066 (0.065)
Epidemiology (d)	0.153* (0.090)	0.165* (0.086)	0.165* (0.086)
Etiology (d)	−0.040 (0.089)	−0.034 (0.088)	−0.034 (0.088)
Genetics (d)	−0.069 (0.062)	−0.080 (0.061)	−0.080 (0.061)
Immunology (d)	0.122 (0.103)	0.101 (0.101)	0.101 (0.101)
Metabolism (d)	0.008 (0.058)	−0.001 (0.058)	−0.001 (0.058)
Mortality (d)	0.088 (0.088)	0.086 (0.088)	0.086 (0.088)
Pathology (d)	−0.016 (0.060)	−0.002 (0.060)	−0.002 (0.060)
Physiopathology (d)	0.123 (0.121)	0.116 (0.122)	0.116 (0.122)
Prevention and control (d)	0.107 (0.097)	0.096 (0.096)	0.096 (0.096)
Radiography (d)	−0.229* (0.128)	−0.196 (0.130)	−0.196 (0.130)
Radiotherapy (d)	−0.055 (0.132)	−0.038 (0.129)	−0.038 (0.129)
Secondary (d)	−0.072 (0.103)	−0.065 (0.103)	−0.065 (0.103)
Surgery (d)	−0.070 (0.063)	−0.040 (0.063)	−0.040 (0.062)
Therapy (d)	−0.021 (0.069)	−0.008 (0.068)	−0.008 (0.068)
Research organizations (d)	Included	Included	Included
Journals (d)	Included	Included	Included
Constant	1.146*** (0.087)	1.110*** (0.085)	
RMSE	1.057	1.051	1.051

*Note*: Robust standard errors are clustered at the journallevel and are reported in parentheses.

*p < .1; **p < .05; ***p < .001.

*Source*: Authors' elaboration.

**FIGURE 2:**
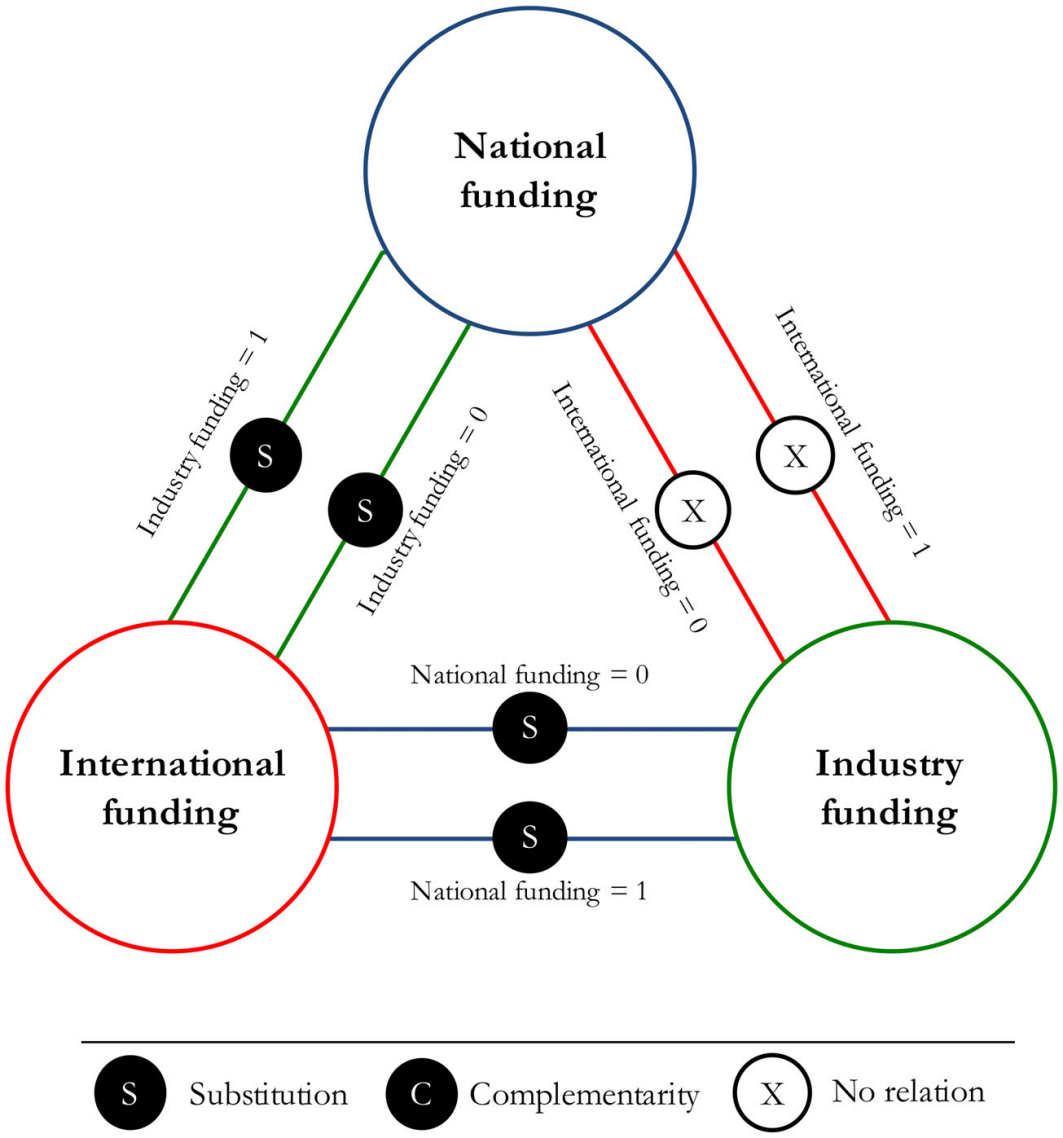
Funding complementarity and substitutability in relation to publications' citations (*Source*: Authors' elaboration)

Following previous research (Antonioli et al., 2017; Ballot et al., 2015; Mohnen & Röller, 2005), we used the coefficients from Model 4 to test for funding complementarity and substitutability according to supermodularity theory. Table 4 summarizes the conditional tests between mutually exclusive combinations of funding types when the number of citations a publication received is considered as a performance measure, while Figure 2 summarizes the findings of the analysis. We found evidence of substitutability (submodularity) between national and international funding—more precisely, there is strong evidence of substitutability when there is no industrial funding supporting a publication, while substitutability is relatively weak if industrial funding is also acknowledged in a publication. Ceteris paribus, when a publication is produced with the support of national (international) funding, introducing international (national) funding is associated with a lower marginal return on the number of citations than introducing only the use of international (national) funding support in the production of a publication. In the same vein, the analysis suggests that international and industrial funding sources are substitutes no matter the presence or absence of national funding. National and industry funding, in contrast, are neither complements nor substitutes.

**TABLE 4:** Testing conditional complementarity/substitutability between mutually exclusive combinations of funding types in relation to a publication's number of citations

Complementarity (>) or substitutability (<)	Restriction	Complementarity/substitutability test	
		Wald test	Coefficient of the linear combination
National/international funding			
$\theta_{110}+\theta_{000}-\theta_{100}-\theta_{010}<>0$	No industry funding	28.91***	−0.395*** (0.073) Substitutability
$\theta_{111}+\theta_{001}-\theta_{101}-\theta_{011}<>0$	With industry funding	3.49*	−0.391* (0.209) Substitutability
National/industry funding			
$\theta_{101}+\theta_{000}-\theta_{100}-\theta_{001}<>0$	No international funding	2.06	−0.195 (0.136) None
$\theta_{111}+\theta_{010}-\theta_{110}-\theta_{011}<>0$	With international funding	1.06	−0.191 (0.185) None
International/industry funding			
$\theta_{011}+\theta_{000}-\theta_{010}-\theta_{001}<>0$	No national funding	14.09***	−0.542*** (0.144) Substitutability
$\theta_{111}+\theta_{100}-\theta_{110}-\theta_{101}<>0$	With national funding	10.73***	−0.538*** (0.164) Substitutability

*Note*: The null hypothesis (H_0_) of the Wald test is that the linear combination of the coefficients is equal to 0. Given that we tested for a linear combination at a time and that we estimated robust standard errors clustered at the journal level (1,317 journals), the *F* statistic has 1 numerator and 1,316 denominator degrees of freedom, hence the corresponding values are 2.71, 3.85, and 6.65 for 0.1, 0.05, and 0.01 levels of significance, respectively.

*p < .1; **p < .05; ***p < .01.

*Source*: Authors' elaboration.

We also examined whether our results are potentially affected by unobserved heterogeneity bias by using the diagnostic test developed by Oster (2019). The method proposed by Oster (2019) enables us to examine how large the influence of selection on unobservables relative to the selection on observables would need to be to nullify the effect of a variable of theoretical interest. We performed Oster's (2019) test on the different combinations of funding types (F100, F010, F001, F110, F101, F011, and F111) by using the “psacalc” command in Stata 17.^6^ This analysis revealed that selection on unobservables would need to be from 1.5 to about 8.6 times stronger than selection on observables, depending on the specific combination of funding types, to nullify the corresponding coefficient. These results suggest that unobserved heterogeneity does not fundamentally bias the direction of the relationships we observe.

## 5 DISCUSSION AND CONCLUSIONS

Our study provides two main contributions. First, we contribute to the stream of research examining the relationship between research funding and outputs (e.g., Defazio et al., 2009; Jacob & Lefgren, 2011). In line with previous studies (e.g., Boyack & Jordan, 2011; Gök et al., 2016), our paper provides further evidence that publications supported by external funding are likely to be associated with a higher number of citations than publications that received no external support. Yet we also demonstrated that this relationship exists for all types of funding sources we examined (i.e., national, international, and industry) as well as for all combinations of these sources.

Second, our paper represents the first attempts to investigate complementarity and substitutability between research funding sources at the level of the single research output. Prior research has explored the question of whether funding sources are complements or substitutes by examining the use of different funding by research organizations and scientists (e.g., Blume‐Kohout et al., 2009; Hottenrott & Lawson, 2017). Exploring this issue at the level of the publication provides an additional perspective on the phenomenon since a large majority of publications are produced with the support of multiple funders (Yan et al., 2018). Thus, there are likely to be several reasons for the co‐occurrence of funders' support in publications, such as single research groups that have benefited from the support of multiple funders simultaneously or in series, or otherwise instances where multiple research groups have collaborated to produce publications, with each acknowledging their own funders in the publications they co‐author. Our results show that national and international funding sources are likely to be observed as jointly supporting publications—suggestive of complementarity as defined by co‐occurrence. Crucially though, when the number of citations is considered, we found substitutability between national and international sources as well as between international and industry funding. We do not find evidence to support complementarity (supermodularity) between funding types at the publication level. Although publications supported by any external funding are likely to be associated with a higher number of citations than those with no external funding support, the combinations of different types of funding do not provide evidence of the emergence of synergistic effects. This may suggest the rise of coordination challenges when funding sources of different types with their own project‐level objectives are added into the research process as well as the presence of decreasing returns in the relationship between funding and the impact of the supported research piece (e.g., Mongeon et al., 2016). However, this does not exclude the possibility that funding types may be complementary when other dimensions of performance are considered such as stimulating collaborative activities between research groups and institutions.

Our analysis presents a number of limitations, which we argue are opportunities for further research on the role of research funding in research systems. First, our study proxies research support by using funding information as acknowledged in publications. While publications are only one of the outputs scientists produce from the support of external funding, funding acknowledgments have important limitations (Aagaard et al., 2021; Rigby, 2013). One limitation is a lack of information about the amount of financial support scientists received or how much of this funding was used to produce the considered publication—a research project could lead to multiple publications as well as to other forms of output. This limitation results from the limited availability of funding data capable of comprehensively covering funding systems and linking research funding data to outputs. Although funding data are increasingly available through databases such as SCOPUS, Web of Science, and MEDLINE/PubMed, research has also suggested that these databases miss a large variety of relatively “small” funders that populate funding systems (Grassano et al., 2017) and considerable coverage issues emerge in the case of historical analysis (Liu, 2021). A more in‐depth analysis of funding complementarity and substitutability requires major institutional efforts to provide researchers with comprehensive access to funding data. In this regard, one relevant example is the Research Portfolio Online Reporting Tools of the US National Institutes of Health, which links grants with individual scientists, research host organizations, publications, patents, and clinical studies. Yet, this is limited to NIH grants and to the United States. In the same vein, access to more granular data on funding would provide scope to explore the complementarity and substitutability phenomenon building on different classifications of funding sources. While the objective of our classification of funding was to examine complementarity and substitutability as a result of UK‐based authors accessing funding opportunities within/beyond the UK research system and in the private sector, more granular data could provide opportunities to assess funding complementarity and substitutability in terms of competitive versus noncompetitve funding as well as across different funding mechanisms.

Second, our paper examines funding complementarity and substitutability considering publications and academic impact (citation count) as the unit of analysis and indicator of research performance, respectively. However, funding complementarity and substitutability can occur within and across the different layers of the research activity—organizations, individuals, and research outputs. Our study is therefore also a call for more multilevel studies examining funding and research systems. In the same vein, funding complementarity and substitutability can emerge in relation to different dimensions of research performance. For example, in line with emerging research investigating the alignment between funding and societal needs (e.g., Ciarli & Ràfols, 2019; Confraria & Wang, 2020), the evaluation of funding complementarity and substitutability can be extended to dimensions of performance such as societal impact, interdisciplinarity, or translational research, thus increasing our understanding of which mixes of funding sources are more likely to contribute to system‐level goals.

Finally, our analysis focused on one research domain in one country in a given year. This research design allowed us to collect data on funding acknowledgements with a high level of accuracy (compared to some other approaches) and to have clear geographical‐, domain‐, and temporal‐boundary conditions for interpretation purposes, especially if one considers that the issue of funding complementarity and substitutability has been overlooked by extant research. It is also true that different interactions among funding sources may emerge in other research domains and systems. Similarly, the cross‐sectional structure of our data limits the possibility of exploring causal links among the variables of theoretical interest in a systematic manner as well as of examining how changes in research funding mechanisms and systems impact complementarity and substitutability among funding sources. Notwithstanding the challenges associated with access to funding data as discussed above, these limitations suggest that to increase our understanding of when and if synergies among funding sources emerge there is a need for more research efforts that build on global and longitudinal data samples covering multiple disciplinary domains. In the same vein, combining longitudinal data on funding with in‐depth qualitative approaches (e.g., interviews with research teams) would allow us to shed light on how researchers combine different types of funding sources and what mechanisms contribute to explain the dynamics of this process, hence potentially the emergence of complementarity and substitutability effects.

In summary, the design of policy actions and funding instruments capable of addressing the major pressures faced by national research funding systems requires a better understanding of the complex dynamics between funding and research activity. Our study contributes to this by providing evidence on the complementarity and substitutability between funding sources of different types that jointly support publications.

## Supporting information


**Table S1.** Descriptive statistics (*N* = 7,439).
**Table S2.** Correlation matrix (*N* = 7,439).
**Table S3.** Regression results on the likelihood of reporting at least one external funding source in publications and on the number of external funding sources reported in publications (*N* = 7,439).

## Endnotes

^1^
One could argue that complementarity and substitutability can be also examined by testing for the inequalities $\Pi \left(1,1\right)\geq \Pi \left(1,0\right)$ and $\Pi \left(1,1\right)\geq \Pi \left(0,1\right)$. While such an empirical strategy would provide evidence of whether publications supported by two funding types are of higher academic impact than publications supported by one funding type, it does not enable us to examine the emergence of synergies (if any) among funding types, which is core to the supermodularity theory and to the objective of our paper.

^2^
Stringer et al. (2010) argued that the logarithm of the number of citations is “a typical value for the number of citations that a paper will ultimately receive” (p. 1384). It is also worth noting that using the number of citations as dependent variable and the Poisson and Negative Binomial as econometric models produce results that are qualitatively similar to those obtained by using the logarithm of the number of citations as dependent variable and an OLS econometric model (Table 3). Yet the OLS model provides a better fit to our data according to the AIC and BIC indicators.

^3^
Our sample of publications includes 1,317 journals—41.5% of these are represented by only one publication in the sample. The top 1% journals by number of publications account for 18.6% of the sample of publications.

^4^
In our econometric specification, we attempt to control for the so‐called “Matthew effect,” which in our case would imply that publications get cumulative citations as a function of the status/prestige of their authors, the organizations to which the authors are affiliated or the journal in which the paper is published. At the journal level, our models include fixed effects for the top 1% journals by number of publications in the sample and our estimates are based on robust standard errors clustered at the level of the journal. These contribute to controlling for the Matthew Effect that is attached to journals (see Larivière & Gingras, 2010). At the organizational level, our models include organizational fixed effects for those research organizations that contributed to at least 1% of the publications in the sample. At the author level, the control variable on the number of authors involved in a publication contributes to account for the “network component” of the Matthew Effect (Wang, 2014). We would argue that this econometric specification is likely to leave the remaining dimensions of the Matthew Effect to exert only residual effects.

^5^
All coefficients associated with the variables representing any combination of funding types are significantly higher (*p* < .05) than the coefficient associated to the variable representing no funding support, that is, “F000 (None).”

^6^
We also multiplied the *R* ^2^ value (0.425) of a model equivalent to Model 4 in Table 3 but with the constant term by the heuristic value of 1.3. Oster (2019) suggested that this heuristic defines the likely maximum variance that the model would explain in the absence of omitted variable bias.
