# Supplementary material for: Do funding sources complement or substitute? Examining the impact of cancer research publications
Source: J Assoc Inf Sci Technol. 2022 Nov 19;74(1):50–66. doi: 10.1002/asi.24726 (PMC10099239; doi:10.1002/asi.24726)
Supplement: Supplementary file 1 — Table S1. Descriptive statistics (N = 7,439). Table S2. Correlation matrix (N = 7,439). Table S3. Regression results on the likelihood of reporting at least one external funding source in publications and on the number of external funding sources reported in publications (N = 7,439). [file ASI-74-50-s001.docx]

# Supplemental Material

## 1. Descriptive statistics

Table S1 and S2 report the descriptive statistics and correlation matrix of all the variables included in our econometric specifications. It is worth noting that the correlation matrix does not suggest major collinearity issues that may affect our econometric estimation. Although there is a relatively high correlation between the *Article (d)* and *Review (d)* (ρ = -0.681) and *Internationality ratio* and *Affiliations ratio* (ρ = 0.750), the econometric results presented in the paper are qualitatively the same if one of these variables is excluded from the models.

Table S1. Descriptive statistics (N = 7,439).

| **Variables** | **Mean** | **S.D.** | **Min** | **Max** |
| --- | --- | --- | --- | --- |
| 1. External funding | 1.575 | 3.207 | 0.000 | 78.00 |
| 2. External funding (d) | 0.517 | 0.500 | 0.000 | 1.000 |
| 3. National funding (d) | 0.343 | 0.475 | 0.000 | 1.000 |
| 4. International funding (d) | 0.235 | 0.424 | 0.000 | 1.000 |
| 5. Industry funding (d) | 0.092 | 0.289 | 0.000 | 1.000 |
| 6. Citations | 3.038 | 1.379 | 0.000 | 8.629 |
| 7. Research variety | 1.072 | 0.591 | 0.000 | 4.000 |
| 8. Research specificity | 4.521 | 1.895 | 1.000 | 9.000 |
| 9. Article (d) | 0.719 | 0.450 | 0.000 | 1.000 |
| 10. Review (d) | 0.153 | 0.360 | 0.000 | 1.000 |
| 11. Number of references | 34.17 | 35.29 | 0.000 | 678.0 |
| 12. Number of authors | 6.940 | 5.958 | 1.000 | 45.00 |
| 13. Internationality ratio | 0.374 | 0.258 | 0.024 | 2.000 |
| 14. Affiliations ratio | 0.540 | 0.306 | 0.038 | 4.000 |
| 15. Research domains (d) | 18 dummy variables | | | |
| 16. Research organisations (d) | 26 dummy variables | | | |
| 17. Journals (d) | 14 dummy variables | | | |

*Source: Authors’ elaboration.*

## 2. Exploratory analysis

Table S3 outlines which covariates are associated with the reporting of funding sources in publications. We first explored the extent to which our independent variables are associated with the number of funding sources that supported a publication – Models 1 and 2 report the results of the Poisson and Negative Binomial estimations, respectively. We then examined the likelihood that a publication is supported by at least one external source of funding – Models 3 and 4 report the results of the Probit and Logit estimations, respectively.

Table S2. Correlation matrix (N = 7,439).

| **Variables** | **1** | **2** | **3** | **4** | **5** | **6** | **7** |
| --- | --- | --- | --- | --- | --- | --- | --- |
| 1. External funding | - |  |  |  |  |  |  |
| 2. External funding (d) | 0.474 | - |  |  |  |  |  |
| 3. National funding (d) | 0.373 | 0.699 | - |  |  |  |  |
| 4. International funding (d) | 0.477 | 0.536 | 0.109 | - |  |  |  |
| 5. Industry funding (d) | 0.272 | 0.308 | 0.017 | 0.073 | - |  |  |
| 6. Citations | 0.237 | 0.403 | 0.262 | 0.269 | 0.180 | - |  |
| 7. Research variety | 0.009 | -0.012 | -0.008 | -0.023 | 0.015 | 0.016 | - |
| 8. Research specificity | -0.015 | -0.067 | -0.053 | -0.039 | -0.004 | -0.081 | 0.115 |
| 9. Article (d) | 0.144 | 0.255 | 0.173 | 0.167 | 0.058 | 0.185 | 0.001 |
| 10. Review (d) | -0.060 | -0.080 | -0.041 | -0.076 | -0.008 | 0.154 | -0.011 |
| 11. Number of references | 0.109 | 0.183 | 0.120 | 0.137 | 0.067 | 0.391 | -0.001 |
| 12. Number of authors | 0.508 | 0.360 | 0.166 | 0.374 | 0.233 | 0.395 | 0.043 |
| 13. Internationality ratio | -0.134 | -0.224 | -0.272 | -0.039 | -0.002 | -0.174 | -0.037 |
| 14. Affiliations ratio | -0.043 | -0.132 | -0.241 | 0.022 | 0.057 | -0.039 | -0.021 |
| **Variables** | **8** | **9** | **10** | **11** | **12** | **13** | **14** |
| 9. Article (d) | 0.101 | - |  |  |  |  |  |
| 10. Review (d) | -0.121 | -0.681 | - |  |  |  |  |
| 11. Number of references | -0.140 | -0.149 | 0.400 | - |  |  |  |
| 12. Number of authors | 0.076 | 0.279 | -0.162 | 0.063 | - |  |  |
| 13. Internationality ratio | -0.135 | -0.367 | 0.203 | 0.050 | -0.398 | - |  |
| 14. Affiliations ratio | -0.124 | -0.262 | 0.163 | 0.081 | -0.166 | 0.750 | - |

*Source: Authors’ elaboration.*

The econometric results suggest that publications focussed on specific cancer issues (*Research specificity*) are less likely to be supported by one or more funding sources, while articles or reviews are more likely to receive support from multiple funders than other types of documents. The number of references reported in a publication is also positively related to the presence of external funding.

When examining the team of authors listed in a publication, the higher the number of authors, the higher is the likelihood that the publication was produced with the support of one or more funding sources. The econometric estimation also suggests that research teams that are based in multiple organisations (*Affiliation ratio*) are negatively associated with publications reporting support of at least one funder. This may suggest that teams in the same organisations simultaneously access multiple funding sources.

Finally, the analysis suggests the presence of domain-specific patterns of external support to research. Publications on the topics of genetics, immunology, and metabolism are more likely to be supported by one or more external funding sources, while publications on topics including complications, diagnosis, radiography, secondary, and surgery, are less likely to report external funding.

Table S3. Regression results on the likelihood of reporting at least one external funding source in publications and on the number of external funding sources reported in publications (N = 7,439).

| **Independent variables** | **(1) External**  **funding**  (Poisson) | **(2) External**  **funding**  (Neg. Bin.) | **(3) External**  **funding (d)**  (Probit) | **(4) External**  **funding**  (Logit) |
| --- | --- | --- | --- | --- |
| Research variety | -0.009 (0.061) | 0.020 (0.056) | -0.020 (0.055) | -0.050 (0.092) |
| Research specificity | -0.031*** (0.011) | -0.053*** (0.011) | -0.054*** (0.010) | -0.094*** (0.016) |
| Article (d) | 0.717*** (0.141) | 0.691*** (0.124) | 0.759*** (0.065) | 1.260*** (0.114) |
| Review (d) | 0.372** (0.156) | 0.317** (0.141) | 0.342*** (0.089) | 0.535*** (0.158) |
| Number of references | 0.004*** (0.001) | 0.006*** (0.001) | 0.005*** (0.001) | 0.010*** (0.001) |
| Number of authors | 0.060*** (0.004) | 0.076*** (0.006) | 0.070*** (0.007) | 0.132*** (0.013) |
| Internationality ratio | 0.027 (0.172) | 0.211 (0.157) | 0.156 (0.140) | 0.375 (0.242) |
| Affiliations ratio | -0.042 (0.179) | -0.179 (0.130) | -0.431*** (0.100) | -0.781*** (0.169) |
| Blood (d) | -0.075 (0.124) | -0.241^†^ (0.137) | -0.261 (0.177) | -0.387 (0.298) |
| Complications (d) | -0.724*** (0.141) | -0.697*** (0.141) | -0.566*** (0.105) | -0.929*** (0.183) |
| Diagnosis (d) | -0.273*** (0.085) | -0.297*** (0.087) | -0.275*** (0.076) | -0.462*** (0.128) |
| Drug therapy (d) | -0.055 (0.084) | -0.032 (0.085) | 0.048 (0.081) | 0.090 (0.136) |
| Epidemiology (d) | 0.120 (0.117) | 0.062 (0.113) | 0.157 (0.124) | 0.292 (0.199) |
| Etiology (d) | 0.171^†^ (0.098) | 0.118 (0.112) | 0.118 (0.131) | 0.182 (0.217) |
| Genetics (d) | 0.164** (0.071) | 0.278*** (0.067) | 0.423*** (0.083) | 0.722*** (0.141) |
| Immunology (d) | 0.549*** (0.196) | 0.406*** (0.116) | 0.352** (0.140) | 0.537** (0.237) |
| Metabolism (d) | 0.304*** (0.085) | 0.346*** (0.075) | 0.451*** (0.092) | 0.789*** (0.154) |
| Mortality (d) | 0.106 (0.175) | 0.093 (0.143) | 0.062 (0.120) | 0.138 (0.212) |
| Pathology (d) | 0.008 (0.069) | 0.001 (0.068) | -0.080 (0.070) | -0.136 (0.117) |
| Physiopathology (d) | 0.116 (0.147) | 0.187 (0.167) | 0.218 (0.165) | 0.350 (0.278) |
| Prevention and control (d) | -0.162 (0.103) | -0.092 (0.118) | 0.136 (0.133) | 0.218 (0.220) |
| Radiography (d) | -0.420** (0.213) | -0.422** (0.211) | -0.412*** (0.150) | -0.648*** (0.249) |
| Radiotherapy (d) | -0.121 (0.161) | -0.117 (0.155) | -0.169 (0.156) | -0.266 (0.257) |
| Secondary (d) | -0.531*** (0.159) | -0.576*** (0.172) | -0.408*** (0.126) | -0.664*** (0.211) |
| Surgery (d) | -0.708*** (0.214) | -0.722*** (0.149) | -0.726*** (0.086) | -1.228*** (0.149) |
| Therapy (d) | -0.197 (0.127) | -0.136 (0.123) | -0.246*** (0.091) | -0.419*** (0.155) |
| Research organisations (d) | Included | Included | Included | Included |
| Journals (d) | Included | Included | Included | Included |
| Constant | -0.837*** (0.162) | -1.049*** (0.146) | -1.020*** (0.112) | -1.841*** (0.194) |
| Log likelihood | -12826.8 | -11029.6 | -3769.7 | -3738.1 |
| (Wald)χ^2^ | 9930.3*** | 2903.6*** | 2644.8*** | 27.98.1*** |
| Pseudo R^2^ | 0.279 | 0.116 | 0.260 | 0.266 |

Notes: *p<0.10, ** p<0.05, *** p<0.01. Robust standard errors are clustered at the journal level and are reported in parentheses. *Source: Authors’ elaboration.*
